# Association between periodontitis and uric acid levels in blood and oral fluids: a systematic review and meta-analysis

**DOI:** 10.1186/s12903-023-02900-8

**Published:** 2023-03-28

**Authors:** Lu-wen Ye, Li Zhao, Ze-song Mei, Ying-hong Zhou, Ting Yu

**Affiliations:** 1 Department of Periodontics, The Affiliated Stomatology Hospital of Guangzhou Medical University, Guangdong Engineering Research Center of Oral Restoration and Reconstruction, Guangzhou Key Laboratory of Basic and Applied Research of Oral Regenerative Medicine, No.195 Dongfeng Road West, Guangzhou, 510182 China; 2 Department of Prosthodontics, Guanghua School of Stomatology, Hospital of Stomatology, Sun Yat-Sen University & Guangdong Provincial Key Laboratory of Stomatology, Guangzhou, 510056 China; 3 School of Dentistry, Faculty of Health and Behavioural Sciences, The University of Queensland, Brisbane, QLD 4006 Australia

**Keywords:** Gout, Hyperuricemia, Periodontitis, Purine, Uric acid

## Abstract

**Background:**

Uric acid, a formerly-known antioxidant that has recently been linked to numerous inflammatory diseases as a pro-inflammatory and -oxidative mediator in pathological conditions. It is imperative to reassess the association between periodontitis and uric acid locally and systematically. The aim of this systematic review was to systemically evaluate the association between periodontitis and the uric acid (UA) levels in blood, saliva and gingival crevicular fluid (GCF).

**Methods:**

Relevant clinical studies up to January 28, 2023 were identified and retrieved from electronic databases including PubMed, Scopus, EMBASE and Web of Science, with periodontitis, uric acid, hyperuricemia and gout as the keywords. The weighted (*WMD*) or standardized mean difference (*SMD*) was calculated using fixed- or random-effect models. Methodological heterogeneity was assessed.

**Results:**

Sixteen eligible observational studies and one RCT were enrolled, which included 1354 patients with periodontitis and 989 controls. Three sample types for UA detection were involved, including blood (*n* = 8), saliva (*n* = 9) and GCF (*n* = 1). Meta-analysis demonstrated an enhanced plasma UA concentration (*WMD* = 1.00 mg/dL, 95% *CI* 0.63 to 1.37, *P* < 0.001) but a decreased salivary UA level (*SMD* = -0.95, 95% *CI* -1.23 to -0.68, *P* < 0.001) in periodontitis versus control. Statistical heterogeneity among the plasma- and saliva-tested studies were moderate (*I*^2^ = *58.3%, P* = *0.066*) and low (*I*^2^ = 33.8%, *P* = 0.196), respectively.

**Conclusions:**

Within the limitations of the enrolled studies, it seems that there is an association between periodontitis and increased blood UA and decreased salivary UA. (Registration no. CRD42020172535 in Prospero).

**Supplementary Information:**

The online version contains supplementary material available at 10.1186/s12903-023-02900-8.

## Introduction

Periodontitis is a multifactorial chronic inflammatory condition caused by an imbalanced interaction between periodontal microbiota and host inflammatory response, which seems to have a bidirectional link with systemic inflammatory diseases [1–3]. The host immune response to periodontal infection can be modified by many genetic and environmental factors, among which gene polymorphisms (e.g., *IL1*) and chronic non-infectious diseases (e.g., diabetes, obesity, etc.) play a significant role [1, 4, 5]. Severe periodontitis affects 23.6% of the global population [6]. In addition to causing tooth loss and a decline in quality of life, periodontitis imposes an enormous socioeconomic burden [7]. Impaired immune and metabolic response induced by periodontal pathogens is the critical feature in the pathogenesis of periodontitis [8, 9]. However, the underlying mechanisms remain unclear, making early prevention challenging.

Oxidative stress has been considered as an important mechanism in the progression of periodontitis [10]. It is formed when the reactive oxygen species overproduced by immune cells when infections cannot be neutralized by the antioxidant defense system, causing lipid, protein and DNA damage [11]. Historically, uric acid (UA) was regarded as an important radical scavenger among the antioxidant pools. However, its anti-oxidative roles appear to be restricted to hydrophilic environments only [12]. Recent studies have identified hyperuricemia (i.e., blood UA levels > 6.5 mg/dL) as risk factors for a variety of inflammatory conditions such as gout, diabetes, metabolic syndrome and cardiovascular diseases [13–15]. Specifically, UA can exhibit pro-oxidative effects in certain environments. For instance, UA can react with peroxynitrite to form radicals [16, 17]. It can also enhance intracellular superoxide production by elevating nicotinamide adenine dinucleotide phosphate (NADPH) oxidase activity [18]. Hence, the current consensus acknowledges that a pathological elevation of UA levels (i.e., hyperuricemia) represents a pro-inflammatory, -oxidative and -osteoclastic state [12].

UA was identified as an anti-oxidative parameter in previous research [19, 20] on periodontitis. Alterations of UA levels in blood have been associated with the presence or severity of periodontitis [21, 22]. Interventions with urate-lowering drugs have shown beneficial effects on animals with periodontitis [23, 24]. However, a re-assessment of their relationship is urgently required because the results of relevant studies are highly contradictory. Firstly, it was discovered that blood UA levels in periodontitis patients were either upregulated, downregulated or unchanged in comparison to controls [25–27]. UA levels in the saliva of periodontitis patients were either reduced or unchanged [28, 29]. Secondly, the presence of periodontitis was associated with an increase in UA levels in the blood but a decrease in saliva UA levels [30]. Thirdly, periodontal treatment increased salivary UA levels but decreased blood UA levels compared to baseline [31]. Integrating the contradictory data would therefore necessitate a systematic review of previous findings. In addition, it would be useful to answer an unresolved question of whether hyperuricemia and periodontitis may be linked [24, 31, 32]. The present systematic review and meta-analysis focuses on the question of whether periodontitis patients have altered UA levels in blood, saliva, and gingival crevicular fluid (GCF) compared to controls.

## Materials and methods

The systematic review and meta-analysis were registered in PROSPERO (no. CRD42020172535) and prepared in accordance with the Preferred Reporting Items for Systematic Reviews and Meta-analyses (PRISMA) statement [33]. The present study adhered to the PECO principles: P (population) was participants in systemic health or with gout/hyperuricemia but without systemic complications/comorbidities; E (exposure) was patients with periodontitis; C (comparison) was periodontally healthy controls; and O (outcome) was UA levels in blood/saliva/GCF.

### Eligibility criteria

The studies should be cohort/cross-sectional/case–control study, or randomized/non-randomized controlled trials. For interventional clinical trials, the baseline data before intervention were deemed as information originated from observational studies. Case reports/series, animal/in-vitro studies, narrative/systematic reviews, conference abstracts, editorials, letters and comments were excluded. The following were the selection criteria for full-text analysis.

### Definition of periodontitis and periodontal health control

Plaque-induced periodontal destruction should be measured by periodontal probing or radiographs to diagnose periodontitis. From an epidemiological standpoint, the periodontal healthy control concerned in the present study would include not only clinical periodontal health but also mild (Stage I) or localized periodontitis.

#### Inclusion criteria

- a) Periodontitis should be clearly defined or stated;
- b) periodontitis and control groups should have UA levels in blood, saliva or GCF measured;
- c) participants in systemic health or with gout/hyperuricemia but without systemic complications (e.g., renal dysfunction including abnormal glomerular filtration rate);
- and d) participants in interventional studies should have baseline/pre-treatment UA levels recorded.

#### Exclusion criteria

- a) studies without periodontitis patients or control groups;
- b) female subjects during pregnancy;
- c) subjects with potential comorbidities shared by hyperuricemia and periodontitis, such as cardiovascular diseases, diabetes, osteoporosis, chronic kidney disease, metabolic syndrome and obesity [31];
- d) subjects with other potential conditions associated with altered purine/UA metabolism such as inflammatory bowel diseases [34, 35], hyperparathyroidism [36] and vitamin D deficiency [37];
- e) subjects with significant systemic diseases or conditions such as cancers, liver cirrhosis, organ transplantation, etc.;
- and f) subjects receiving antibiotics or anti-inflammatory or urate-lowering drugs or have had periodontal treatment in the past three months.

### Information sources and search strategy

Highly sensitive electronic search was conducted in four databases, including PubMed, Scopus, Web of Science and Embase with no language restriction (update to January 28, 2023). The following search model was constructed using Boolean operators. For exposure, “periodontal diseases” and “periodontitis” were used, while “apical periodontitis” was excluded. Regarding UA-tested samples, the keywords “blood”, “serum”, “plasma”, “circulation”, “GCF”, “gingival crevicular fluid” and “saliva” were used. As for outcome, the keywords “UA”, “urate”, “purine”, “hyperuricemia”, “gout” and “antioxidant” were used. The advanced search was based on each database-specific search strategies (see the Supporting Information). The search terms employed were either medical subject headings (MeSH) terms or keywords classified under general category (title/abstract/keywords).

### Study selection

Initial assessment of titles and abstracts was conducted by two independent reviewers (L.Y. and L. Z.), followed by full-text screening of the eligible articles. The disagreement was discussed until consensus was reached or the supervisor arbitrated (T. Y.). During the process, any study that failed to meet the eligibility criteria was excluded and the reason was formally recorded in detail. Inter-examiner agreement for abstract review and full-text screening was assessed using the *κ* test (*κ* values > 0.75 and < 0.4 indicated high and low consistency, respectively) [38].

### Quality assessment and quality of evidence

Newcastle–Ottawa Scale (NOS) and the Agency for Healthcare Research and Quality (AHRQ) methodology checklist were used to assess the methodological quality of case–control studies and cross-sectional studies, respectively. As for randomized controlled trials, only the baseline data before intervention were collected and the research design was deemed as an observational one. Namely, RCTs were also evaluated by NOS during quality assessment. Quality assessment was conducted by two reviewers independently (L. Y. and L. Z.). Inter-examiner agreement for quality assessment were assessed by the *κ* test.

The total quality score on the Newcastle–Ottawa Scale ranged from 0 to 9 stars (7 to 9, 5 to 6 and < 5 stars indicated high, moderate and low quality, respectively) [39]. Regarding ARHQ, studies with 8 to 11 points, 4 to 7 points, and 0 to 3 points, respectively, were deemed to be of high, moderate and low quality [40]. Disputes would be discussed with a third reviewer (T. Y.).

The certainty of evidence was evaluated following the Grade of Recommendations Assessment, Development and Evaluation (GRADE) method with GRADEprofiler (v 3.6, the GRADE working group) [41, 42]. The evidence quality of each outcome was rated as high, moderate, low and very low.

### Data extraction

Once these studies were identified, two reviewers independently (L. Y. and L. Z.) extracted the information including bibliometric information (the names, e-mail addresses and institutions of authors, publication date and journals of articles, etc.), characteristics of study design (study place/type, diagnostic criteria of periodontitis/control groups and sample size, etc.), demographics of participants (sex, age, race and smoking habit [43, 44], periodontal parameters (probing pocket depth, gingival/plaque index, bleeding on probing, clinical attachment loss, radiological alveolar bone loss, etc.), collection methods for saliva (resting/stimulated) or blood (plasma/serum) or GCF (paper strip/point), detection methods for UA (enzyme-based colorimetric method or gas chromatography/mass spectrometer, etc.), statistics for UA levels (means and standard deviations (*SD*s)/errors (*SE*s)) in periodontitis and control groups. Data extraction forms were cross-checked to verify accuracy and consistency of the extracted data. All data were checked by the third author (T. Y.) and disagreements were resolved by discussion. Three emails were sent to the corresponding authors of the included articles to request the raw data, which include the gender and age of the participants. Unanswered or undelivered emails were regarded as having no response.

### Data conversion and preprocessing

The UA concentrations may be recorded in mg/dL, µmol/L, mmol/L or relative units. The unit reported in this systematic review was mg/dL (1 mg/dL = 59.48 µmol/L) [45]. According to the new classification for periodontal diseases [46], chronic and aggressive periodontitis are no longer distinguished from one another, and the data presented here were compiled in accordance with this classification. The data of UA in periodontitis at different stages or in a single type of sample collected using different methods were also combined based on the means, *SD*s and sample size of the subgroups. If some studies reported *SEs* instead of *SD*s, the former was converted to the latter (*SD* = *SE* × $\surd n$) [47]. If the data of UA in a study were shown in bar charts without detailed values, the heights of three repeats were measured using a digital ruler (v1.8.0, Image J, National Institutes of Health, Bethesda, MD, USA) to determine the absolute values.

### Statistical analyses

Statistical analyses were performed using a commercial software (v 14.0, STATA, Stata Corporation, College Station, TX). The UA levels are presented as means ± *SDs*. The distribution of potential confounding variables (such as gender and age) that may influence the UA levels was compared [48]. Meta-analyses are displayed as forest plots. It would be calculated as weighted mean differences (*WMDs*) or standardized mean differences (*SMDs*) for continuous outcomes. Statistical heterogeneity was estimated by Cochrane’s *Q* test and Higgins’s *I*^2^ test (*I*^2^ = 0, no heterogeneity; *I*^2^ < 50%, low heterogeneity; *I*^2^ < 75%, moderate heterogeneity; *I*^2^ > 75%, high heterogeneity) [49]. If a statistically significant heterogeneity was found, a random-effect model was used; otherwise, a fixed-effect model was applied. A subgroup analysis was conducted based on the collection methods of testing samples (i.e., serum vs. plasma). *P* < 0.05 was considered statistically significant. Publication bias was assessed by Egger’s tests [50]. If publication bias was detected, the Duval and Tweedie trim-and-fill method was used to make adjustments [51]. If statistical heterogeneity was detected, a sensitivity analysis was conducted by excluding each study individually to determine whether the heterogeneity changed significantly.

## Results

### Study selection

**Fig. 1:**
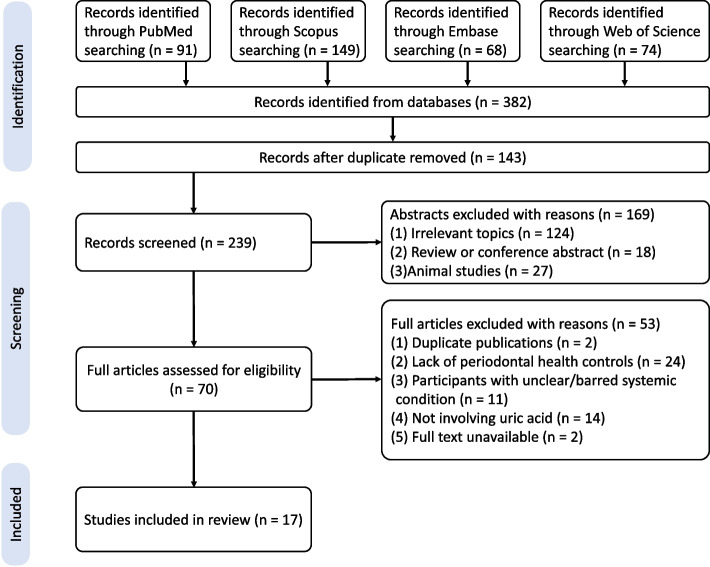
PRISMA flow diagram of the selection process

A total of 382 potentially eligible records were found through a highly sensitive electronic search, of which 239 were included for abstract review and 70 for full-text evaluation. Finally, 17 articles were retained for systematic review [22, 25–29, 52–62]. Additionally, the *κ* value of agreement between the two examiners for the abstract review (*κ* = 0.81, 95% *CI* 0.74 to 0.88) and full-text review (*κ* = 0.87, 95% *CI* 0.76 to 0.98) demonstrated excellent consistency. Table 1 provided a summary of the characteristics of the 17 included studies. Figure 1 demonstrated the selection procedure for included. The reasons for excluded studies are recorded in Table S1.

**Table 1:** Detailed characteristics of included studies

Citations	Study location	Funding source	Study type	Diagnosis of periodontitis	Statement on periodontal controls	Sample size [P (M/F) vs. C (M/F)]	Age (years; P vs. C) ^†^	Periodontal parameters (mm, P vs. C) ^†^	Sample/Collection method	Detection method for UA	UA levels (P vs. C) ^†^	Quality assessment
**Gharbi et al., 2019** [26]	Africa (Tunisia)	College	Case control	AAP criteria	Periodontal health	80 (33/47) vs. 50 (25/25)	42 ± 13.6 vs. 44.8 ± 12.6	PPD: 5.3 (2.5–7.5) vs. 1 (0.5–2)	Blood/Plasma	EBCM	5.22 ± 0.91 vs. 4.13 ± 1.11(mg/dL) ^‡^	NOS:8
**Banu et al., 2015** [25]	Asia (India)	Unknown	Case control	Clinically ≥ 4 teeth in each jaw; PPD ≥ 5 mm; CAL ≥ 4 mm; ≥ 80% BOP + at proximal sites; presence of ABL in ≥ 2 quadrants of the dentition; interproximal ABL ≥ 50%	Without periodontitis	40 (14/26) vs. 20 (9/11)	40–65	PPD: 5.55 ± 0.29 vs. 2.28 ± 0.13	Blood/Plasma	EBCM	5.32 ± 0.95 vs. 4.42 ± 0.68 (mg/dL)	NOS:8
**Mourão et al., 2015** [55]	South America (Brazil)	Unknown	Case control	AAP criteria	PPD < 3 mm in all tooth sites and absence of CAL	20 (8/12) vs. 20 (8/12)	54.3 ± 10.02 vs. 50.2 ± 8.79	NM	Blood/Plasma	EBCM	6.45 ± 1.44 vs. 4.77 ± 0.81 (mg/dL)	NOS:7
**Merle, C.L., et al., 2022** [62]	Europe (German)	University	Cross-sectional	CPITN > 2	CPITN ≤ 2	32 (17/15) vs. 53(25/28)	21.6 ± 3.9 vs. 21.4 ± 3.2	NM	Blood/Plasma	NM	4.76 ± 1.02 vs 4.22 ± 1.25 (mg/dL) ^‡^	AHRQ: 9
**Narendra et al., 2018** [27]	Asia (India)	Unknown	Cross-sectional	AAP criteria	NM	78 (40/38) vs. 50 (33/17)	38.34 ± 12.08 vs. 36.56 ± 6.26	CAL: 4.60 ± 0.51 vs. 1.49 ± 0.25	Blood/Serum	EBCM	5.10 ± 0.31 vs. 5.11 ± 0.54 (mg/dL)	AHRQ:6
**Sreeram et al., 2015** [60]	Asia (India)	Unknown	Cross-sectional	≥ 14 teeth; BOP + at ≥ 30% periodontal sites with PPD = 1–3 mm; BOP + and CAL ≥ 3 mm at ≥ 30% of all sites	≥ 14 teeth; BOP + at < 30% sites with PPD = 1–3 mm; BOP + and CAL ≥ 3 mm at < 30% of all sites	150 (114/36) vs. 150 (120/30)	41.0 ± 12.2 vs. 34.2 ± 12.0	NM	Blood/Serum	EBCM	4.29 ± 1.15 vs. 4.93 ± 0.87 (mg/dL)	AHRQ:7
**Brotto et al., 2011** [52]	South America (Brasil)	University	Case control	≥ 14 teeth including third molars; at least 4 different teeth had at least one site with PPD = 3–5 mm, and at least 4 others different teeth had at least one site with PPD = 6–10 mm; the proportion of all sites were considered to be AL > 2 mm and PPD > 2 mm	≥ 14 teeth including third molars; BOP + at < 30% sites with PPD = 1–3 mm, only 2 isolated sites with PPD = 4 mm and BOP-, and CAL ≥ 3 mm at < 30% of all sites	30 (16/14) vs. 30 (16/14)	46 ± 6 vs. 43 ± 5	PPD: 2.25 (1.39–3.62) vs. 1.00 (1.00–1.40) CAL: 2.58 (1.45–4.50) vs. 1.00 (1.00–2.27)	Blood/Serum	EBCM	4.9 ± 2.1 vs. 4.3 ± 1.7 (mg/dL)	NOS:6
**Tsai et al., 2021** [22]	Asia (China)	National institutions	Cross-sectional	Localized stage II/III periodontitis	Periodontally healthy or stage I periodontitis	295(269/26) vs. 828(726/102)	30.88 ± 5.35 vs. 29.38 ± 5.56	PPD: 3.03 ± 0.04 vs.2.92 ± 0.05 CAL: 3.07 ± 0.06 vs. 2.93 ± 0.06	Blood/Serum	EBCM	6.73 ± 1.45 vs. 6.49 ± 1.41 (md/dL)	AHRQ:7
**Sakanaka et al., 2017** [57]	Asia (Japan)	University	Case control	PISA > 215	PISA < 215	35 vs. 15	NM	PISA: 490.8 (200.5–1238.5) vs. 199.3 (155.5–252.8)	Saliva/Resting	Gas Chromatography—Mass Spectrometry	112.32 ± 66.02 vs. 155.83 ± 82.69 (Intensity)^§?^	NOS:6
**Novakovic et al., 2014** [56]	Europe (Serbia)	National institutions	RCT	≥ 3 teeth per quadrant; at least one pocket PPD > 5 mm with BOP + per quadrant; and ABL > 30%	Periodontally healthy	42 (28/14) vs. 21 (14/7)	39.0 ± 11.81 vs. 35.2 ± 7.1	PPD: 3.38 ± 0.58 vs. 2.11 ± 1.67; CAL: 3.00 ± 1.00 vs. 0	Saliva/Resting	EBCM	153.93 ± 40.88 vs. 198.43 ± 87.73 (relative level)	NOS:6
**Miricescu et al., 2014** [28]	Europe (Romania)	National institutions	Cross-sectional	Gingival inflammation; at least six sites with PPD ≥ 4 mm; and ABL > 30%	NM	25 (11/14) vs. 25 (5/20)	51.26 ± 7.4 vs. 18.66 ± 2.0	PPD: 4.41 ± 0.42 vs. 0	Saliva/Resting	EBCM	2.41 ± 0.27 vs. 3.12 ± 0.85 (mg/mg albumin)	AHRQ:4
**Mathur et al., 2013** [54]	Asia (India)	Unknown	Case control	CPITN > 2	CPITN ≤ 2	30 vs. 10	NM	NM	Saliva/Resting	EBCM	2.34 ± 0.4 vs. 5.19 ± 0.8 (relative level)	NOS:4
**Fatima G et al., 2016** [53]	Asia (India)	Unknown	Case control	PPD = 6 mm or presence of CAL	No periodontal pockets as assessed by Williams periodontal probe	10 (4/6) vs. 10 (4/6)	46.30 ± 8.62 vs. 27.30 ± 4.37	NM	Saliva/Resting	EBCM	3.01 ± 0.68 vs. 5.39 ± 1.49 (mg/dL)	NOS:7
**Sharma et al., 2018** [59]	Asia (India)	Nil	Cross-sectional	Russell’s periodontal index and a panoramic radiograph	Russell’s periodontal index, and a panoramic radiograph	25 vs. 25	34.32 vs. 30.68	NM	Saliva/Resting	EBCM	1.95 ± 0.42 vs. 3.72 ± 1.02(mg/dL)	AHRQ:7
**Senouci et al., 2021** [58]	Africa (Algeria)	University	Case control	stage III– IV, grade C periodontitis	without clinical signs of periodontal disease as measured by PPD or any CAL	29 (6/23) vs. 28 (7/21)	24.06 ± 6.09 vs. 24.73 ± 1.38	PPD: 7 ± 1.68 vs. 1.7 ± 0.3; CAL: 7.81 ± 1.79 vs. 1.4 ± 0.2	Saliva/Resting	EBCM	1.43 ± 0.93 vs. 2.78 ± 1.60(mg/dL)	NOS:8
**Priya, K.L., et al., 2022** [61]	Asia (India)	University	Case control	Stage II/III, grade B periodontitis	PPD ≤ 3 mm, without attachment loss or radiographic bone loss	20 vs. 20	30–65	PPD: 5.10 ± 0.26 vs. 1.36 ± 0.27 CAL: 5.47 ± 0.23 vs. 1.35 ± 0.27	Saliva/Resting	EBCM	5.64 ± 4.32 vs. 21.49 ± 10.01(mg/dL)	NOS: 6
**Diab-Ladki et al., 2003** [29]	Asia(Lebanon)	National institutions	Case control	Severe periodontitis (with tooth mobility, gingival recession and up to one-half of ABL)	Apparently healthy gingiva	17 vs. 20	30–45	NM	Saliva/Stimulated	EBCM	2.41 ± 2.32 vs. 2.68 ± 2.46 (mg/dL) ^‡§?^	NOS:2
**Narendra et al., 2018** [27]	Asia (India)	Unknown	Cross-sectional	AAP criteria	NM	78 (40/38) vs. 50 (33/17)	38.34 ± 12.08 vs. 36.56 ± 6.26	CAL: 4.60 ± 0.51 vs. 1.49 ± 0.25	GCF/Paper strip	EBCM	4.87 ± 0.36 vs. 5.11 ± 0.53 (mg/dL)	AHRQ:6

The AAP criteria for diagnosis of periodontitis was defined as ≥ 2 interproximal sites with CAL ≥ 3 mm and ≥ 2 interproximal sites with PD ≥ 4 mm (not on the same tooth) or one site with PPD ≥ 5 mm [63, 64]; PISA is a calculated index based on BOP^+^, CAL and gingival recession to reflect periodontal inflammation. PISA score for an individual tooth = percentage of BOP^+^ sites × (attachment loss surface area—recession surface area). The PISA scores of total teeth in a mouth were summed to get a total PISA value for a patient [65]. The CPITN is an index with five degrees according to BOP, presence of dental calculus and PPD (0, normal; 1, gingivitis with BOP^+^; 2, presence of calculus; 3, PPD ≥ 3.5 mm; and 4, PPD ≥ 5.5 mm) [66]. †, the data of periodontal parameters, age and UA levels are presented as means ± standard deviations or medians (interquartile range);‡, For uric acid, 1 mg/dL p;’ = 59.48 µmol/L; §, Standard errors were converted to standard deviations; ?, Data of UA in bar charts without numerical display were measured using a digital ruler; *AAP* American academy of periodontology, *ABL* Alveolar bone loss, *AHRQ* Agency for healthcare research and quality, *BOP* Bleeding on probing, *CAL* Clinical attachment loss, *CPITN* Community periodontal index of treatment needs, *EBCM* Enzymatic colorimetric methods, *GCF* Gingival crevicular fluid, *NM* Not mentioned, *NOS* Newcastle–Ottawa scale, *M/F* Male/female, *P vs. C* periodontitis vs. control, *PPD* Probing pocket depth, *PISA* Periodontal inflamed surface area

### General characteristics of included studies

The detailed characteristics of the 17 included studies are presented in Table 1. The 17 identified studies were published between 2003 and 2022. Ten studies were conducted in Asia [22, 25, 27, 29, 53, 54, 57, 59–61], three in Europe [28, 56, 62], two in South America [52, 55] and two in Africa [26, 58]. There were ten case–control studies [25, 26, 29, 52–55, 57, 58, 61], six cross-sectional studies [22, 27, 28, 59, 60, 62] and one RCT [56]. A total of 2343 participants were included (989 patients with periodontitis vs. 1354 healthy controls). The participants generally aged between 18 and 65. The majority of the studies (11/17) have a balanced gender distribution between periodontitis and control groups [22, 25–28, 52, 53, 55, 56, 58, 60]. The sample sizes in the studies ranged from 20 to 1123. Three sample types were involved for UA detection including blood (eight studies) [22, 25–27, 52, 55, 60, 62], saliva (nine studies) [28, 29, 53, 54, 56–59, 61] and GCF (one study) [27]. One study involved two sample types, i.e., blood and GCF [27]. Fifteen studies detected the UA levels with enzymatic colorimetric methods [22, 25–29, 52–56, 58–61], while one analyzed the UA content using gas chromatography-mass spectrometry [57]. Most of the studies (10/17) were funded by non-profit organizations. Specifically, one study was funded by a college [26], five by universities [52, 57, 58, 61, 62] and four by national institutions [22, 28, 29, 56].

### Definition of periodontitis and control

Regarding the exposure factor, nine studies provided clear but different criteria for defining periodontitis. Among them, three studies used the criteria of American Academy of Periodontology (AAP) in 1999 [26, 27, 55], three used the new classification for periodontal diseases in 2018 [22, 58, 61], and two used Community Periodontal Index of Treatment Needs (CPITN) [54, 62]. In addition, one study distinguished periodontitis from the control group according to the periodontal inflamed surface area (a secondary index calculated from clinical attachment loss, gingival recession and bleeding on probing) [57]. The remaining eight studies were either self-defined (7/8) [25, 28, 52, 53, 56, 59, 60] or included only severe periodontitis without clear diagnostic criteria (1/8) [29].

In the included studies, the definitions of the controls for periodontitis were also diverse. Six studies claimed clinical periodontal health as controls [25, 26, 29, 55, 56, 61]. Six studies provided clear definitions of controls. Specifically, two studies used CPITN ≤ 2 (0, normal; 1, gingivitis with bleeding on probing; 2, presence of calculus) as controls [54, 62], while one used periodontal inflamed surface area (PISA) < 215 [57]. In addition, two studies allowed the presence of localized periodontitis in the control groups [52, 60], and another one combined healthy periodontium with stage I periodontitis [22]. Three of the remaining studies provided a self-defined statement on controls [53, 58, 59], while two made no mention of controls [27, 28].

Gender and age were two potential confounding variables for the subgroup analysis, which could not be conducted due to missing data. Furthermore, smokers were clearly rejected in the majority of studies (13/17) [25–28, 52–54, 56–61]. Three studies failed to specify whether or not they included smokers [29, 55, 62]. Only one study considered body morphology [62], where body mass index was slightly unbalanced, but body weight was comparable between periodontitis and control groups.

### Quality assessment and evidence quality

The two reviewers assessed the risk of bias with a high degree of agreement (*κ* = 0.85, 95% *CI* 0.77 to 0.94). The results of the assessment of the risk of bias were shown in Table S2 and S3. Of the ten case–control studies evaluated by NOS, five were of high quality (7 to 8 points) [25, 26, 53, 55, 58], four were of moderate quality (6 points) [52, 57, 61] and two were of low quality (2 and 4 points) (Table S2) [29, 54]. The single RCT assessed by NOS showed moderate quality [56]. The quality of the six cross-sectional studies ranged from moderate (4 to 7 points) [22, 27, 28, 59, 60] to high (9 points) [62] according to the AHRQ checklist (Table S3). The GRADE evidence quality for all outcomes was very low (Table 2).

**Table 2:** The Grading of Recommendations Assessment, Development, and Evaluation (GRADE) assessment for the outcomes

Quality assessment							No of patients		Effect		Quality
**No of studies**	**Design**	**Risk of bias**	**Inconsistency**	**Indirectness**	**Imprecision**	**Other considerations**	**Periodontitis**	**Periodontal healthy**	**Relative (95% CI)**	**Absolute**	
**Blood UA levels (Better indicated by lower values)**											
8	observational studies	serious^1^	serious^2^	no serious indirectness	no serious imprecision	none	725	1201	-	*WMD* 0.50 mg/dL higher (0.06 to 0.93 higher)	⊕ΟΟΟ VERY LOW
**Saliva UA levels (Better indicated by lower values)**											
9	observational studies	serious^1^	no serious inconsistency	no serious indirectness	no serious imprecision	reporting bias	233	174	-	*SMD* -0.95 lower (-1.23 to -0.68 lower)	⊕ΟΟΟ VERY LOW

^1^ The statistical analysis in these studies did not adjust for potential confounding risk factors such as age, gender, smoking condition or body mass index

^2^ Statistical heterogeneity between the studies was high (*I*^2^ = 93.5%)

### Blood UA levels and periodontitis

**Fig. 2:**
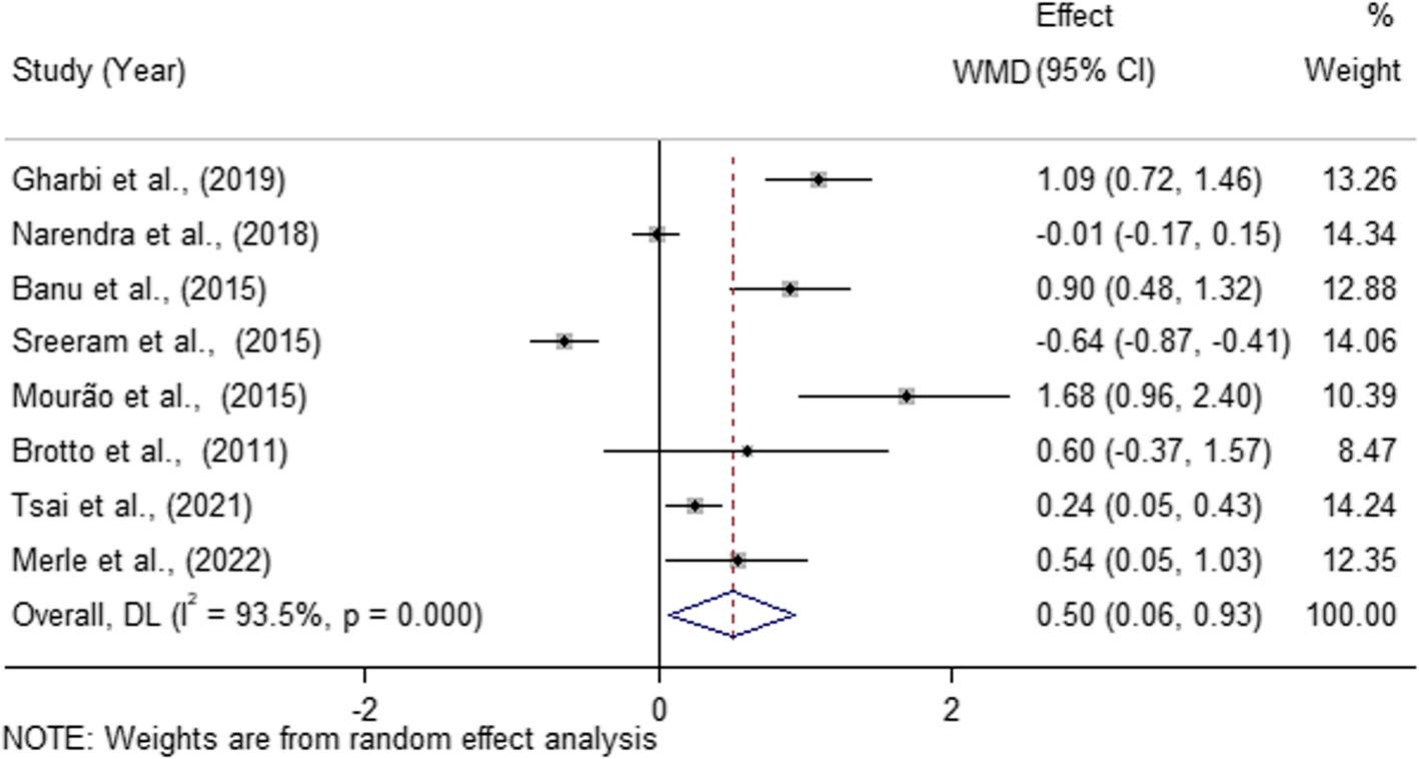
Forest plot for comparing the UA levels in blood in periodontitis vs. control. *CI,* confidence interval; *WMD*, weighted mean difference.

Eight studies (725 periodontitis patients vs. 1201 controls) were included to determine whether periodontitis patients have altered UA levels in their blood compared to control [22, 25–27, 52, 55, 60, 62]. Four of them used plasma and the other four used serum as testing samples. Six studies found increased UA levels in blood in periodontitis compared to controls, while one study found a decrease and the remaining studies found no significant change. Because the enrolled studies utilized a universal unit, the UA concentration in blood can be combined. Consequently, the combined study results were presented as *WMD*. Using a random-effect model, a meta-analysis revealed that patients with periodontitis had a slightly higher UA blood level (*WMD* = 0.50 mg/dL, 95% *CI* 0.06 to 0.93, *P* = 0.025) (Fig. 2). However, the lower limit of the confidence interval was approaching zero. Additionally, the statistical heterogeneity between the studies was substantial (*I*^2^ = 93.5%,* P* < 0.001). The sensitivity analysis failed to identify any discernible effect of individual studies on the pooled risk estimates (Figure S1). Next, subgroup analysis based on blood collection methods was conducted (plasma vs. serum). Compared to controls, periodontitis patients had significantly higher UA levels in plasma (*WMD* = 1.00 mg/dL, 95% *CI* 0.63 to 1.37, *P* < 0.001) but comparable UA levels in serum (*WMD* = -0.04 mg/dL, 95% *CI* -0.47 to 0.39, *P* = 0.847) (Figure S2). In this instance, statistical heterogeneity was low in the plasma subgroup (*I*^2^ = 58.3%, *P* = 0.066), whereas it was high in the serum subgroup (*I*^2^ = 91.6%, *P* < 0.001).

### Salivary UA levels and periodontitis

**Fig. 3:**
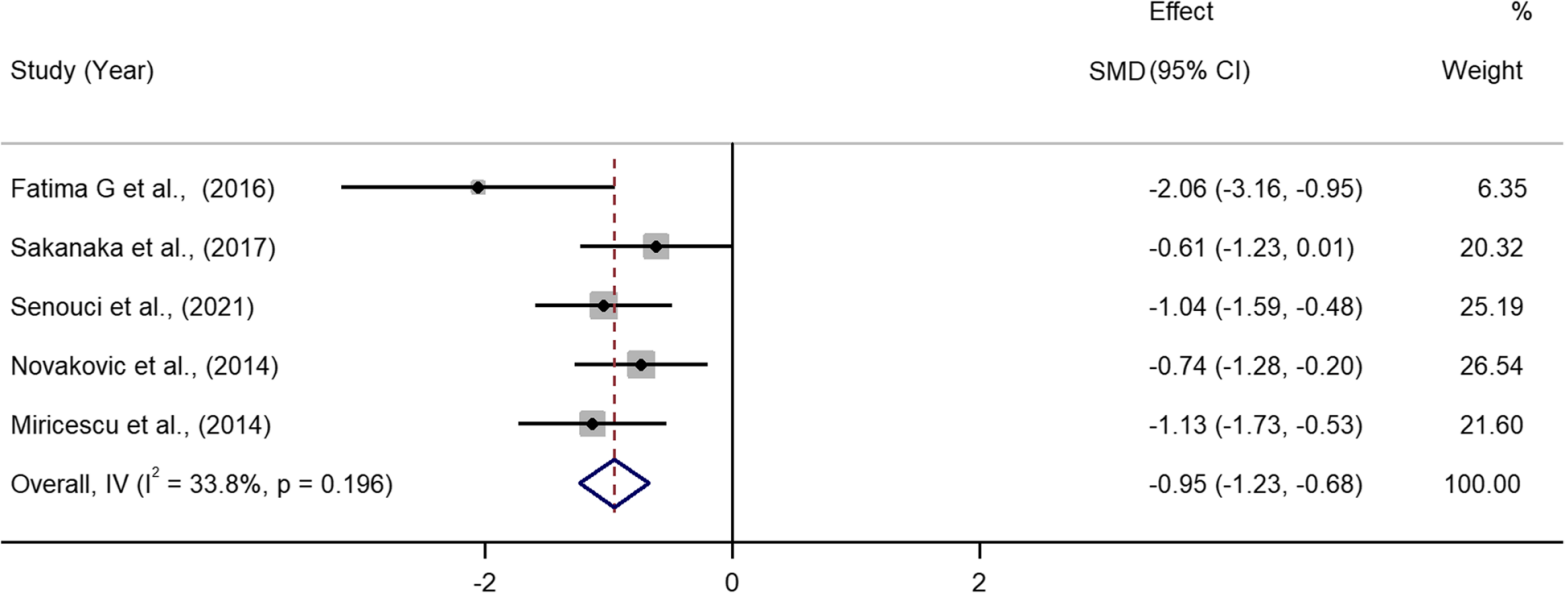
Forest plot for comparing the UA levels in saliva in periodontitis vs. control. *SMD*, standardized mean difference.

The second question is “whether periodontitis patients have altered UA levels in their saliva compared to the control?” Included were nine studies with 233 periodontitis patients and 174 controls [28, 29, 53, 54, 56–59, 61]. In contrast to the content of UA in blood, the content of UA in saliva could not be standardized in the enrolled studies due to the use of various units (five used mg/dL and the other four provided semi-quantitative data). Consequently, the combined study results were presented as *SMD*. In nine studies, salivary UA levels were consistently lower in patients with periodontitis compared to controls. Meta-analysis utilizing a random-effect model demonstrated that salivary UA content was significantly lower in periodontitis than in controls (*SMD* = -1.57, 95% *CI* -2.25 to -0.90, *P* < 0.001) (Figure S3). There was considerable heterogeneity between the studies (*I*^2^ = 88.0%, *P* < 0.001). By eliminating four studies, sensitivity analysis (Figure S4) assisted in achieving a low level of heterogeneity (*I*^2^ = 33.8%, *P* = 0.196) [29, 54, 59, 61]. In this instance, periodontitis was still associated with a decrease in UA levels in saliva relative to the control (*SMD* = -0.95, 95% *CI* -1.23 to -0.68, *P* < 0.001), as determined by a fixed-effect model (Fig. 3).

### Association of UA in GCF with periodontitis

Only one cross-sectional study (78 vs. 50, periodontitis patients vs. controls) reported UA levels in GCF (Table1) [27]. The study was deemed to be of high quality (5 points) (Table S2). In this study, GCF was collected using paper strips, and UA concentration was determined using enzymatic colorimetric methods. Similar to the findings in saliva, the outcome demonstrated a significant decrease 4.93% in UA levels in periodontitis compared controls [4.87 ± 0.36 vs. 5.11 ± 0.53 (mg/dL), *P* < 0.001].

### Publication bias

Publication bias cannot be ruled out given the small number (< 10) of both blood- (*n* = 8) and saliva-tested studies (*n* = 9). Mandatory analysis with Egger's test showed no publication bias in studies detecting blood UA (*P* = 0.118, *n* = 8), but a potential bias in studies involving salivary UA (*P* = 0.012, *n* = 9).

## Discussion

The present study explored the association between oral/blood UA and periodontitis using systematic review and meta-analysis. We discovered a positive correlation between periodontitis and blood UA content. The increased blood UA might result from accelerated purine degradation in both periodontal tissues and systemic organs. Patients with periodontitis exhibit accelerated purine catabolism and enhanced xanthine oxidoreductase expression in the periodontium [30, 67]. Enhanced secretion of UA has been observed in immune cells stimulated by periodontal pathogens [67, 68], and in the gingiva of mice with periodontitis [9]. Given that ubiquitous xanthine oxidoreductase is sensitive to inflammation and oxidative stress [69], periodontitis-induced low-grade systemic inflammation may accelerate purine catabolism in distant organs. For instance, periodontal infection is associated with increased UA levels in the liver and feces in rodents [70, 71]. However, it should be noted that the present study only supported a positive association between blood UA and periodontitis when normouricemia was present. UA has been considered as a potent antioxidant in blood, especially at a physiological level [12, 72, 73]. Nonetheless, such a theory has not been rigorously examined in the field of periodontology. It is unknown whether elevated UA levels in the circulation or periodontal vessels in a state of normouricemia are beneficial or detrimental for periodontitis.

Almost no direct evidence has investigated the relationship between periodontitis and hyperuricemia or gout. One cross-sectional study identified hyperuricemia as a protecting factor for periodontitis [32]. However, the outcome was measured by questionnaire and only represented a retrospective history of periodontitis. Gouty patients generally have increased abundance of periodontal pathogens (e.g., *Prevotella Intermedia*) [74]. Given that the highly prevalent hyperuricemia is considered an emerging risk factor for many inflammatory comorbidities of periodontitis (e.g., cardiovascular disease, diabetes, and chronic kidney disease) [75], the association between hyperuricemia and periodontitis would make for an intriguing research topic. Recent research indicates that UA plays a pathological role in periodontitis. For instance, systemic injection of UA aggravated alveolar bone loss in mice with periodontitis [24]. The urate-lowering drug febuxostat alleviated experimental periodontitis induced by molar ligation in rats [23]. Moreover, non-surgical periodontal treatment for periodontitis appeared to reduce urate levels in the circulation [76]. Taken together, pathologically elevated blood UA appears to contribute to the progression of periodontitis.

Interestingly, the change of UA in saliva shows an opposite trend compared to that in blood in periodontitis patients versus controls. UA has been considered as a major antioxidant in saliva, accounting for ~ 70% of the total antioxidant capacity [77]. However, the levels of antioxidants, including UA, in saliva do not appear to correspond to those in blood [19]. A reduced level of salivary UA in periodontitis may be due to either increased consumption or decreased production. Increased consumption of salivary UA may be a result of the enhanced oxidative stress caused by periodontal infection. In the presence of oxidative stress, UA may be oxidized by reactive oxygen species into allantoin in the absence of uricase [78]. Additionally, UA can serve as a substrate for the synthesis of bacterial components [31], a process that may be accelerated by an increase in dental plaque accumulation. Substances that inhibit the purine oxidation activity of the UA-producing enzyme (i.e., xanthine oxidoreductase) may lead to a decrease in salivary UA production. For instance, there is an increase in the demand of nitrate and its metabolites nitrite and nitric oxide in saliva for bactericidal and anti-inflammatory purposes in periodontitis [79]. These substances may competitively inhibit the purine-oxidizing activity of xanthine oxidoreductase, considering that xanthine oxidoreductase also has a nitrate/nitrite reductase activity [80, 81]. In summary, the mechanisms underlying the reduction of salivary UA in periodontitis are unclear, and further investigation is warranted.

The present systematic review included only one article that found a decreased level of UA in GCF in periodontitis patients. The result was consistent with the findings from some studies that were not included in the review [30, 82]. The changes of UA in GCF resembled those in saliva. Different from saliva that comes from salivary glands, GCF is serum transudate in healthy periodontium or from inflammatory exudate during periodontal diseases [83]. In the context of periodontitis with an increased UA content in the blood, a decrease in the exudation of UA from circulation into GCF was unlikely. A more probable scenario, similar to that of saliva, would be that subgingival microbiota could enhance UA consumption. Moreover, it should be noted that some urate transporters (SLC2A9 and SLC22A12) showed increased gene expressions in gingival tissues from periodontitis patients [84], which could be a potential contributor or confounder for an altered UA concentration in periodontal pockets.

There were several potential confounding variables that could have affected the applicability of this study. The data of sex, age and smoking, which have been associated with hyperuricemia or altered tissular UA in the past studies [85–87], were partly missing. We failed to obtain the original data from the corresponding authors in relevant studies. However, the distribution of age and sex was even in most or all of the studies (age, 6/11; sex, 11/11). Smoking was unlikely to have a significant impact on the results given that most (12/15) of the studies excluded smokers. The data of body mass index (a common cofounding factor), which has been reported to significantly impact blood UA [88, 89], were completely missing. Consequently, it was unclear how much and to what extent body mass index influences the results.

The definition and control of periodontitis could be another source of bias. Periodontitis is frequently defined differently in clinical studies and systematic reviews. It may diminish the comparability of the studies included in the present systematic review. No studies can make a diagnosis without probing depth, clinical attachment loss, or radiographic bone loss, despite the fact that diagnostic thresholds for periodontitis vary. In the majority of the included studies in this review, the diagnosis was based on these periodontal parameters. Specifically, a clinical misdiagnosis regarding whether or not it was periodontitis rarely occurred. Instead, the discrepancies mainly occurred due to the severity and extent of periodontitis. Indeed, the severity of periodontitis appears to be correlated with UA levels in blood or saliva [19, 21, 31, 90]. For instance, patients with severe periodontitis had higher blood UA levels than those with mild or moderate periodontitis [21]. In this context, the meta-analysis tended to homogenize the data, but did not cover up the effect of periodontitis. Another concern involves the definitions of controls. In many studies, the control groups included not only healthy periodontium, but also gingivitis, mild periodontitis and even localized moderate-to-severe periodontitis. This is a common practice in case definitions when conducting clinical research, which could lead to an underestimation of the differences in UA levels between periodontitis and controls. However, the direction of the disparities would not be changed. Future research should utilize a uniform criterion on periodontitis and controls, i.e., the 2018 classifications of periodontal diseases, in order to enhance comparability between included studies.

Methods of sample collection and analysis may be potential confounding variables. Some studies found no difference between plasma and serum UA levels as measured by enzymatic colorimetric methods [91, 92]. As determined by gas chromatography–mass spectrometry in a separated study, the UA concentration in plasma was 1.59 times that in serum [93]. The discrepancy could be attributed to the differences in technological sensitivity [94]. The present study did not calibrate the UA levels in the two types of blood samples because all of the included studies detected the UA content using enzymatic colorimetric methods. Notably, subgroup analysis on studies involving blood UA showed that the statistical heterogeneity was significantly lower in the plasma subgroup than that in serum. A better homogeneity in the plasma subgroup may be partly due to those anticoagulants (e.g., ethylenediamine tetraacetic acid) inhibit xanthine oxidoreductase activity and thus reduce UA production from undesired sources [95], whereas sustained purine degradation may still occur in serum. Another anticoagulant heparin sodium, however, does not appear to affect xanthine oxidoreductase activity [96]. Therefore, plasma with specific anticoagulants may be preferrable to serum for comparing the results of blood UA content across studies. The combined data from studies using plasma would be more reliable from a heterogeneity standpoint than those using serum. In addition, the effect size from studies using plasma was clearly greater than that from all relevant studies using blood regardless of plasma or serum. Another concern would be whether collection methods influence the results of UA levels in saliva. Resting saliva appears to contain more UA than stimulated saliva [77]. The ratios of resting to stimulated salivary UA was found to be approximately 2:1 in both periodontitis patients and healthy controls. In addition, the concluding meta-analysis excluded studies involving stimulated saliva. Thus, the method of saliva collection may not be a significant confounder in the present meta-analysis.

The present systematic review and meta-analysis had some limitations. First, the number and sample size of included studies were limited, particularly those involving saliva and GCF. Even if the 4 studies (all involving saliva) with small sample sizes(i.e., n < 20) were excluded from the meta-analysis [29, 53, 54, 57], the main findings regarding forest plots did not change significantly. The findings should be interpreted with caution until they are confirmed by large-scale studies. Second, the majority of the findings were derived from retrospective studies, which must be confirmed by prospective and interventional studies. Lastly, the raw data for some potential confounding variables (i.e., age, smoking, and body mass index) were unavailable and their effects (especially body mass index) on the results were unknown. It may contribute to statistical heterogeneity in the present meta-analysis. Taken together, high-quality studies, particularly prospective cohort studies and interventional (e.g., periodontal or urate-lowering treatments) studies, are required to elucidate the association between periodontitis and UA in blood and oral fluids.

## Conclusions

**Fig. 4:**
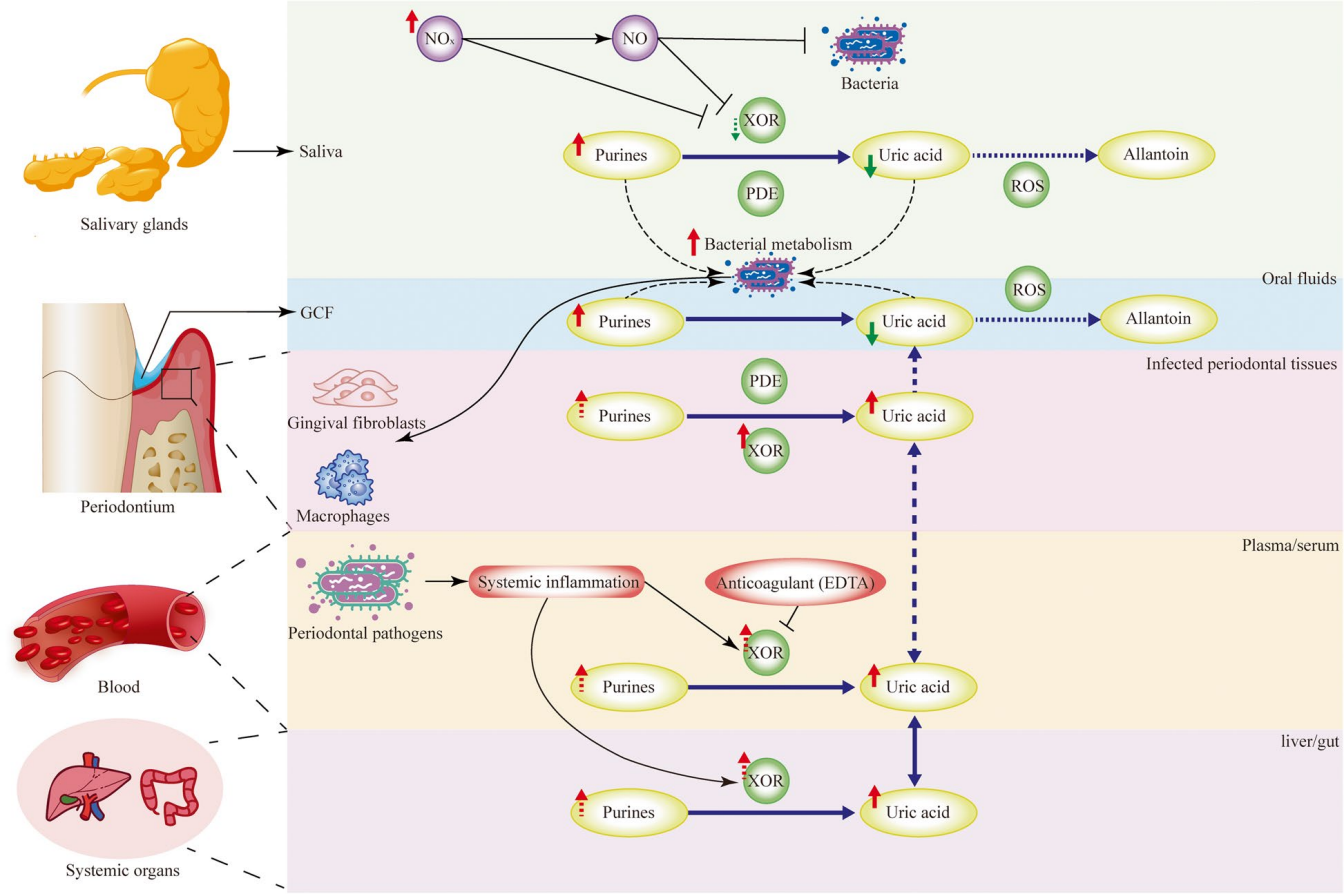
A hypothetical illustration of the differences in uric acid levels between blood, saliva and GCF in periodontitis populations. Saliva and GCF purine levels are found to be elevated in hosts with periodontitis. However, uric acid levels of decrease rather than increase, which may be due to an enhanced uric acid consumption by oral/periodontal bacteria and ROS. Purines in saliva and GCF may also be consumed by XOR-like purine-degrading enzymes that are produced by bacteria. By inhibiting XOR activity, increased levels of nitrate and nitrite produced by salivary glands to combat oral microbiota would reduce the production of uric acid in saliva. In periodontal tissues, circulation and systemic organs (e.g., liver and gut), elevated levels of uric acid have been detected in periodontitis patients or animals, which may be the result of accelerated purine degradation and enhanced XOR activity. The XOR activity in circulation may be increased by periodontitis-related systemic inflammation, but inhibited by anticoagulants such as EDTA. Uric acid may be exchanged between periodontal tissues and systemic organs through circulation. EDTA, ethylenediamine tetraacetic acid; GCF, gingival crevicular fluid; NO_X_, NO_3_^−^ and NO_2_^−^; PDE, purine-degrading enzymes; ROS, reactive oxygen species; XOR, xanthine oxidoreductase.

Within the limitations of the present study, it might be concluded that:

- 1) Periodontitis appears to be associated with an increased blood UA concentration in the context of normouricemia. It remains to be determined whether periodontitis and hyperuricemia/gout are associated.
- 2) In contrast to the change of UA in the blood, the amount of UA in saliva and GCF seems to be decreased in the presence of periodontitis. The potential mechanisms underlying the reversal of changes require additional investigations. Figure 4 depicts a hypothetical representation of the differences between blood, saliva and GCF thorough time.
- And 3) The majority of the findings are based on a small number of observational studies with small sample sizes and substantial methodological heterogeneity, which may compromise the reliability of the conclusions. To further validate the findings, high-quality studies, including large-scale prospective cohort studies and interventional studies, are required.

## Supplementary Information

**Additional file 1**: **Table S1.** The characteristics of excluded studies. **Table S2.** Quality assessment of the included case-control studies with Newcastle-Ottawa Scale. **Table S3.** Agency for Healthcare Research and Quality for risk of bias assessment of the cross-sectional studies. **Figure S1.** Sensitivity analysis of the relationship between periodontitis and controls of UA levels in blood. In these studies, no clearly heterogeneous origin could be found. **Figure S2.** Forest plot comparing UA levels of periodontitis vs. control in plasma/serum subgroups. CI, confidence interval; WMD, weighted mean difference. **Figure S3.** Forest plot comparing the salivary UA levels of periodontitis vs control before sensitivity analysis. There was a high heterogeneity among the studies (I^2^ = 88.6%, P < 0.001). Therefore, sensitivity analysis should be performed to find sources of heterogeneity. SMD, standardized mean difference. **Figure S4.** Sensitivity analysis of the relationship between periodontitis and controls of UA levels in saliva. A sensitivity analysis was performed to explore potential sources of heterogeneity. Statistical heterogeneity was decreased obviously, indicating that they were likely the source of heterogeneity.

## Data Availability

The datasets for this study can be made available on reasonable request to dent_yu@163.com.
