# Supplementary material for: Association between periodontitis and uric acid levels in blood and oral fluids: a systematic review and meta-analysis
Source: BMC Oral Health. 2023 Mar 28;23:178. doi: 10.1186/s12903-023-02900-8 (PMC10045947; doi:10.1186/s12903-023-02900-8)
Supplement: Supplementary file 1 — Additional file 1: Table S1. The characteristics of excluded studies. Table S2. Quality assessment of the included case-control studies with Newcastle-Ottawa Scale. Table S3. Agency for Healthcare Research and Quality for risk of bias assessment of the cross-sectional studies. Figure S1. Sensitivity analysis of the relationship between periodontitis and controls of UA levels in blood. In these studies, no clearly heterogeneous origin could be found. Figure S2. Forest plot comparing UA levels of periodontitis vs. control in plasma/serum subgroups. CI, confidence interval; WMD, weighted mean difference. Figure S3. Forest plot comparing the salivary UA levels of periodontitis vs control before sensitivity analysis. There was a high heterogeneity among the studies (I2 = 88.6%, P < 0.001). Therefore, sensitivity analysis should be performed to find sources of heterogeneity. SMD, standardized mean difference. Figure S4. Sensitivity analysis of the relationship between periodontitis and controls of UA levels in saliva. A sensitivity analysis was performed to explore potential sources of heterogeneity. Statistical heterogeneity was decreased obviously, indicating that they were likely the source of heterogeneity. [file 12903_2023_2900_MOESM1_ESM.docx]

**MATERIALS AND METHODS**

**Search strategies**

Highly sensitive electronic search was conducted from four databases, including the PubMed, Scopus, and Embase (update to January 28, 2023) with no language restriction.

**The search strategy on PubMed**

( ( "uric acid" [Title/Abstract] OR urate [Title/Abstract] OR purine [Title/Abstract] OR hyperuricemia [Title/Abstract] OR gout [Title/Abstract] ) OR (antioxidant [Title/Abstract] AND ( "uric acid" OR urate OR purine OR hyperuricemia OR gout) ) ) AND ( "Periodontal Diseases" [MeSH Terms] OR periodontitis NOT "apical periodontitis" ) AND ( blood [Title/Abstract] OR serum [Title/Abstract] OR plasma [Title/Abstract] OR circulation [Title/Abstract] OR GCF [Title/Abstract] OR "gingival crevicular fluid" [Title/Abstract] OR saliva [Title/Abstract] )

**The search strategy on SCOPUS**

( TITLE-ABS-KEY ( blood OR serum OR plasma OR circulation OR gcf OR "gingival crevicular fluid" OR saliva ) AND DOCTYPE ( ar ) ) AND ( ( ( ALL ( "uric acid" OR urate OR purine OR hyperuricemia OR gout ) AND TITLE-ABS-KEY ( antioxidant ) ) AND DOCTYPE ( ar ) ) OR ( TITLE-ABS-KEY ( "uric acid" OR urate OR purine OR hyperuricemia OR gout ) AND DOCTYPE ( ar ) ) ) AND ( ( TITLE-ABS-KEY ( "periodontal disease" ) OR TITLE-ABS-KEY ( periodontitis ) AND NOT TITLE-ABS-KEY ( "apical periodontitis" ) ) AND DOCTYPE ( ar ) )

**The search strategy on EMBASE**

( 'uric acid':ti,ab,kw OR urate:ti,ab,kw OR purine:ti,ab,kw OR hyperuricemia:ti,ab,kw OR gout:ti,ab,kw ) OR ( ( 'uric acid' OR urate OR purine OR hyperuricemia OR gout ) AND antioxidant:ti,ab,kw ) AND ( ( 'periodontal disease':ti,ab,kw OR periodontitis:ti,ab,kw ) NOT 'apical periodontitis':ti,ab,kw ) AND ( blood:ti,ab,kw OR serum:ti,ab,kw OR plasma:ti,ab,kw OR circulation:ti,ab,kw OR gcf:ti,ab,kw OR 'gingival crevicular fluid':ti,ab,kw OR saliva:ti,ab,kw )

**The search strategy on Web of Science**

( "uric acid" OR urate OR purine OR hyperuricemia OR gout ) AND ( "periodontal disease" OR periodontitis NOT "apical periodontitis" ) AND ( blood OR serum OR plasma OR circulation OR gcf OR "gingival crevicular fluid" OR saliva )

**TABLES**

**Table S1.** The characteristics of excluded studies.

| **Study** | **Year** | **Type of study** | **Reasons for exclusion** |
| --- | --- | --- | --- |
| Almerich-Silla, J M. et al. | 2015 | Cross-sectional study | Not involving uric acid |
| Al-Rawi, N H. et al. | 2017 | Case-control study | Participants with unclear/barred systemic condition |
| Arora, N. et al. | 2013 | Randomized controlled trials study | Lack of periodontal health controls |
| Babaei, H. et al. | 2018 | Randomized controlled trials study | Lack of periodontal health controls |
| Barnes, V M. et al. | 2014 | Cross-sectional study | Not involving uric acid |
| Barnes, V M. et al. | 2009 | Case-control study | Lack of periodontal health controls |
| Batista, E L. et al. | 2010 | Interventional study | Lack of periodontal health controls |
| Botelho, J. et al. | 2020 | Retrospective cohort study | Participants with unclear/barred systemic condition |
| Byun, S H. et al. | 2020 | Cross-sectional study | Lack of periodontal health controls |
| Cao, Y. et al. | 2016 | Cross-sectional study | Participants with unclear/barred systemic condition |
| Chapple, I L C. et al. | 2007 | Prospective cohort study | Lack of periodontal health controls |
| Chapple, I L C. et al. | 1997 | Cross-sectional study | Lack of periodontal health controls |
| Chen, H W. et al. | 2018 | Cross-sectional study | Not involving uric acid |
| Choromańska, M. et al. | 2017 | Case-control study | Participants with unclear/barred systemic condition |
| Fons-Badal, C. et al. | 2019 | Randomized controlled trials study | Lack of periodontal health controls |
| Fornasaro, S. et al. | 2021 | Case-control study | Not involving uric acid |
| Front, E. et al. | 2013 | Interventional study | Lack of periodontal health controls |
| Garg, N. et al. | 2006 | Case-control study | Lack of periodontal health controls |
| Greabu, M. et al. | 2006 | Interventional study | Lack of periodontal health controls |
| Gümüş, P. et al. | 2009 | Case-control study | Lack of periodontal health controls |
| Gumus, P. et al. | 2015 | Case-control study | Not involving uric acid |
| Khodaii, Z. et al. | 2019 | Case-control study | Full text unavailable |
| Konopka, T. et al. | 2007 | Case-control study | Not involving uric acid |
| Lee, C. et al. | 2018 | Prospective cohort study | Lack of periodontal health controls |
| Mashayekhi, F. et al. | 2005 | Case-control study | Not involving uric acid |
| Miricescu, D. et al. | 2011 | Case-control study | Duplicate publication |
| Moore, S. et al. | 1994 | Case-control study | Participants with unclear/barred systemic condition |
| Mourāo, L C. et al. | 2014 | Randomized controlled trials study | Lack of periodontal health controls |
| Munther, S | 2019 | Cross-sectional study | Lack of periodontal health controls |
| Nguyen, T T. et al. | 2017 | Case-control study | Not involving uric acid |
| Novaković, N. et al. | 2013 | Interventional study | Duplicate publication |
| Olszewska-Czyz, I. et al. | 2022 | Cross-sectional study | Lack of periodontal health controls |
| Pulido-Moran, M. et al. | 2017 | Cross-sectional study | Participants with unclear/barred systemic condition |
| Rai, B. et al. | 2011 | Interventional study | Lack of periodontal health controls |
| Sculley, D V. et al. | 2003 | Prospective cohort study | Participants with unclear/barred systemic condition |
| Seydanur et al. | 2019 | Randomized controlled trials study | Lack of periodontal health controls |
| Sezer, U. et al. | 2016 | Case-control study | Not involving uric acid |
| Shetty, M S. et al. | 2018 | Case-control study | Participants with unclear/barred systemic condition |
| Shetty, M. et al. | 2012 | Case-control study | Full text unavailable |
| Sincar, C D. et al. | 2017 | Case-control study | Lack of periodontal health controls |
| Su, H. et al. | 2009 | Cross-sectional study | Not involving uric acid |
| Summers, C J. et al. | 1968 | Cross-sectional study | Lack of periodontal health controls |
| Tamaki, N. et al. | 2015 | Cross-sectional study | Not involving uric acid |
| Tartaglia, G M. et al. | 2017 | Cross-sectional study | Lack of periodontal health controls |
| Hu, S A. et al. | 2022 | Cross-sectional study | Lack of periodontal health controls |
| Chabuk, M I. et al. | 2021 | Case-control study | Lack of periodontal health controls |
| Tu, Y K. et al. | 2013 | Prospective cohort study | Participants with unclear/barred systemic condition |
| Vernerov, A. et al. | 2021 | Case-control study | Lack of periodontal health controls |
| Yoshino, F. et al. | 2012 | Cross-sectional study | Not involving uric acid |
| Ziebolz, D. et al. | 2007 | Cross-sectional study | Participants with unclear/barred systemic condition |
| Zipkin, I. et al. | 1964 | Case-control study | Participants with unclear/barred systemic condition |
| Chen, S., et al., | 2023 | Case-control study | Not involving uric acid |
| Toczewska, J., et al., | 2022 | Case-control study | Not involving uric acid |

**Table S2.** Quality assessment of the included case-control studies with Newcastle-Ottawa Scale.

| Studies | Selection | | | | | Comparability | | Exposure | | | Final Score |
| --- | --- | --- | --- | --- | --- | --- | --- | --- | --- | --- | --- |
|  | **Is the case definition adequate?** | | **Representativeness of the cases?** | **Selection of controls** | **Definition of controls** | **Comparability of cases and controls on the basis of the design or analysis** | | **Ascertainment of exposure** | **Same method of ascertainment for cases and controls** | **Non-response rate** |  |
|  |  |  |  |  |  | **Main factor**^†^ | **Additional factor**^‡^ |  |  |  |  |
| Gharbi et al., 2019 | | 1 | 1 | 0 | 1 | 1 | 1 | 1 | 1 | 1 | 8 |
| Sakanaka et al., 2017 | | 1 | 1 | 0 | 1 | 0 | 0 | 1 | 1 | 1 | 6 |
| Banu et al., 2015 | | 1 | 1 | 0 | 1 | 1 | 1 | 1 | 1 | 1 | 8 |
| Diab-Ladki et al., 2003 | | 0 | 0 | 0 | 1 | 0 | 0 | 0 | 0 | 1 | 2 |
| Mourão et al., 2015 | | 0 | 1 | 0 | 1 | 1 | 1 | 1 | 1 | 1 | 7 |
| Mathur et al., 2013 | | 0 | 1 | 0 | 1 | 0 | 0 | 0 | 1 | 1 | 4 |
| Brotto et al., 2011 | | 0 | 1 | 1 | 1 | 1 | 0 | 1 | 1 | 0 | 6 |
| Fatima G et al., 2016 | | 0 | 1 | 1 | 1 | 1 | 0 | 1 | 1 | 1 | 7 |
| Senouci et al., 2021 | | 1 | 1 | 1 | 0 | 1 | 1 | 0 | 1 | 1 | 8 |
| Novakovic et al., 2014 | | 1 | 1 | 0 | 1 | 1 | 1 | 1 | 1 | 1 | 8 |
| Priya, K.L., et al., 2022 | | 1 | 0 | 1 | 1 | 0 | 0 | 1 | 1 | 1 | 6 |

†, Gender was selected as the main factor; ‡, Age was considered as an additional factor.

**Table S3**. Agency for Healthcare Research and Quality for risk of bias assessment of the cross-sectional studies.

|  | **Narendra et al., 2018** | **Miricescu et al., 2014** | **Sreeram et al., 2015** | **Sharma et al., 2018** | **Tsai et al., 2021** | **Merle, C.L., et al., 2022** |
| --- | --- | --- | --- | --- | --- | --- |
| **Define the source of information** | Yes | Yes | Yes | Yes | Yes | Yes |
| **List inclusion and exclusion criteria for exposed and unexposed subjects (cases and controls) or refer to previous publications** | Yes | No | Yes | Yes | Yes | Yes |
| **Indicate time period used for identifying patients** | No | No | Yes | No | Yes | Yes |
| **Indicate whether or not subjects were consecutive if not population-based** | No | Yes | Yes | Yes | No | Yes |
| **Indicate if evaluators of subjective components of study were masked to other aspects of the status of the participants** | Yes | Yes | Yes | Yes | Yes | Yes |
| **Describe any assessments undertaken for quality assurance purposes** | No | No | No | No | No | No |
| **Explain any patient exclusions from analysis** | Yes | No | Yes | Yes | Yes | Yes |
| **Describe how confouding was assessed and/or controlled** | Yes | No | No | Yes | Yes | Yes |
| **If applicable, explain how missing data were handled in the analysis** | No | No | No | No | No | Yes |
| **Summarize patient response rates and completeness of data collection** | Yes | Yes | Yes | Yes | Yes | Yes |
| **Clarify what follow-up, if any, was expected and the percentage of patients for which incomplete data of follow-up was obtained** | No | No | No | No | No | No |
| **Total score** | 6 | 4 | 7 | 7 | 7 | 9 |

**FIGURES**

**
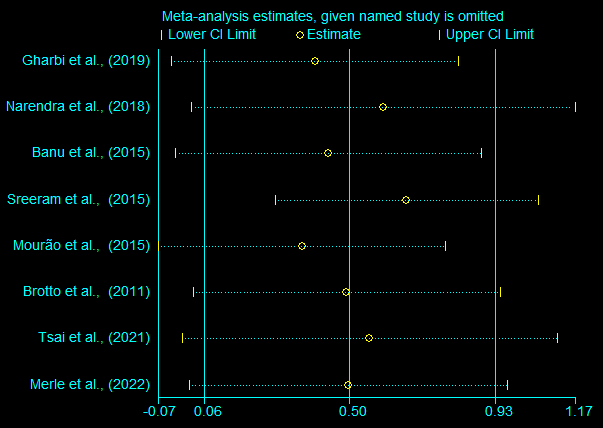
**

**Figure S1.** Sensitivity analysis of the relationship between periodontitis and controls of UA levels in blood.

In these studies, no clearly heterogeneous origin could be found.

**
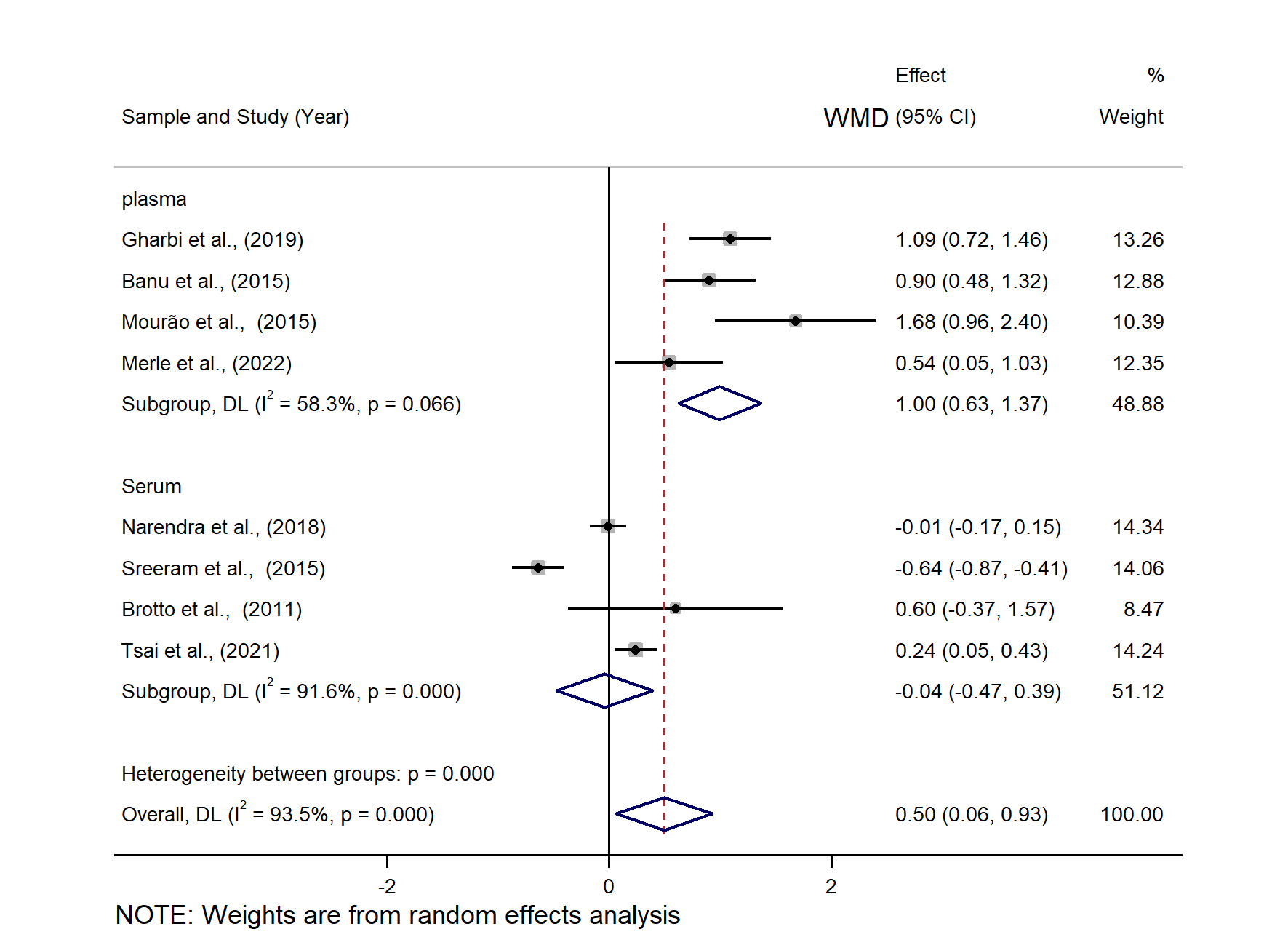
**

**Figure S2.** Forest plot comparing UA levels of periodontitis vs. control in plasma/serum subgroups.

*CI,* confidence interval; *WMD*, weighted mean difference.

**
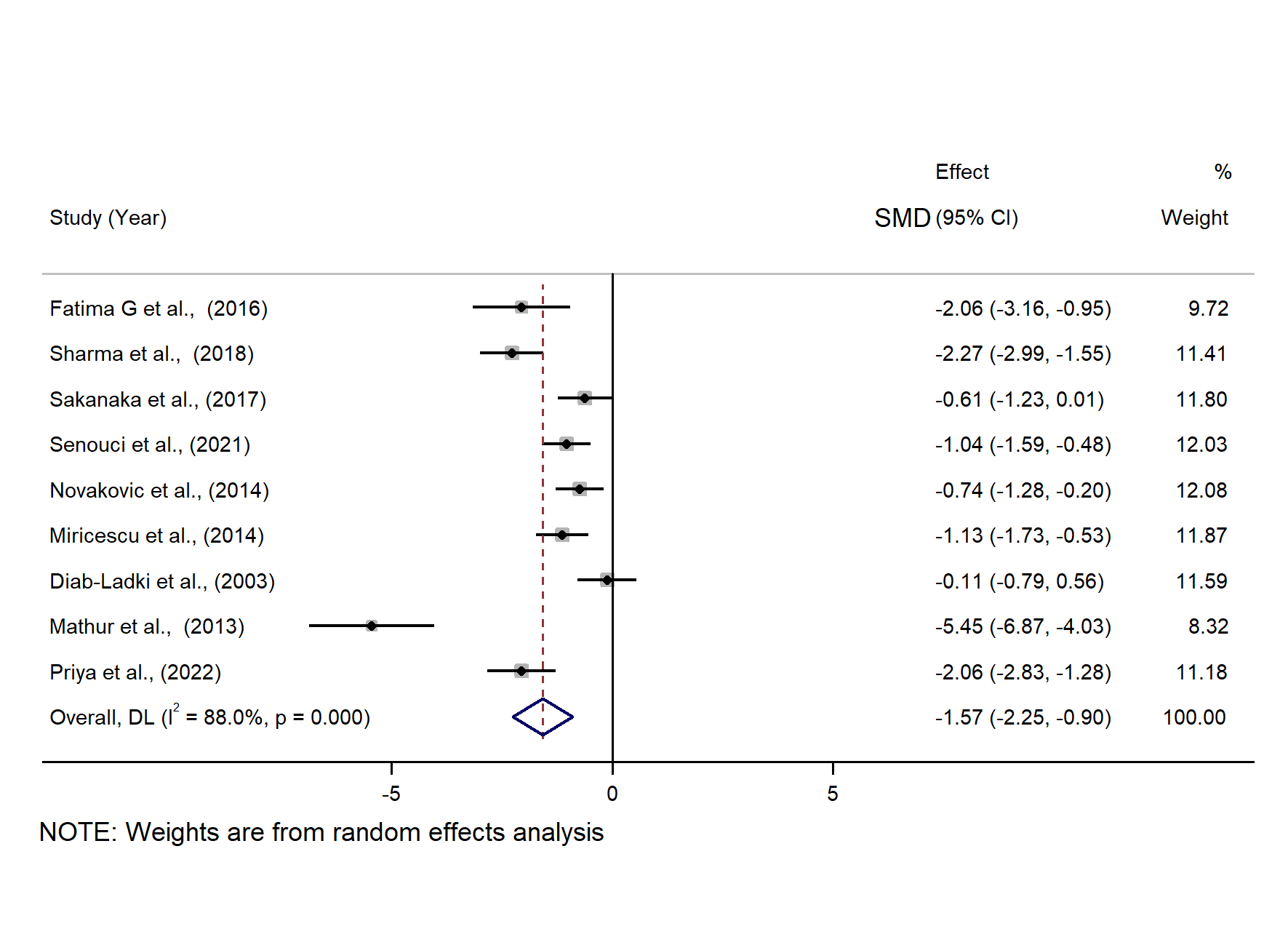
**

**Figure S3.** Forest plot comparing the salivary UA levels of periodontitis vs control before sensitivity analysis.

There was a high heterogeneity among the studies (*I*^2^ = 88.6%, *P* < 0.001). Therefore, sensitivity analysis should be performed to find sources of heterogeneity. *SMD*, standardized mean difference.

**
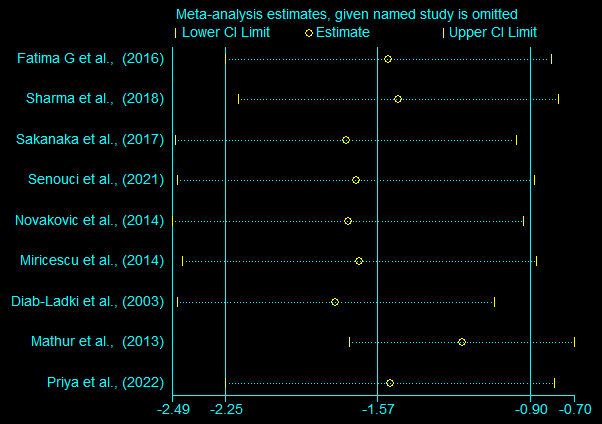
**

**Figure S4.** Sensitivity analysis of the relationship between periodontitis and controls of UA levels in saliva.

A sensitivity analysis was performed to explore potential sources of heterogeneity. Statistical heterogeneity was decreased obviously, indicating that they were likely the source of heterogeneity.
